# Impact of the COVID-19 lockdown on screen media use in patients referred for ADHD to child and adolescent psychiatry: an introduction to problematic use of the internet in ADHD and results of a survey

**DOI:** 10.1007/s00702-021-02332-0

**Published:** 2021-04-22

**Authors:** Anna Maria Werling, Susanne Walitza, Renate Drechsler

**Affiliations:** 1 Department of Child and Adolescent Psychiatry and Psychotherapy, Centre for Child and Adolescent Psychiatric Research, Neuropsychology, University Hospital of Psychiatry Zurich, University of Zurich, Eisengasse 16, 8008 Zurich, Switzerland; 2 Neuroscience Center Zurich, University of Zurich and ETH Zurich, Zurich, Switzerland

**Keywords:** Attention deficit hyperactivity disorder, Problematic use of the internet, COVID-19, Adolescents, Gaming, Social media

## Abstract

The COVID-19 outbreak and lockdown have been associated with multiple consequences for mental health, including an excessive and potentially harmful increase in screen media use. The specific consequences for children, adolescents and young adults with ADHD are still unknown. In the first part of this study, a short review of problematic use of the internet (PUI) in ADHD is presented, showing that patients with ADHD are at risk for different aspects of PUI, such as excessive gaming or problematic social media use. In the second part, we report original data of an online survey on screen media use before, during and after the lockdown completed by parents of children and adolescents clinically referred for ADHD. Parents rated children’s/adolescents’ media-related behavior and media time on a new screening questionnaire for PUI. Each item was rated three times, referring to the observed behavior before, during and 1–2 months after the lockdown. *N* = 126 parents of patients referred for ADHD aged 10–18 years participated in the study. Total media time increased by 46% during the lockdown and did not completely return to pre-Corona levels afterwards. Patients with difficulties concentrating, high irritability or deterioration of ADHD problems under lockdown spent more time with screen media than those with milder or no such problems. While the effects of the lockdown on screen media use and its negative impact on everyday life appear to be largely reversible, a small proportion of patients with ADHD apparently continue to show increased media use.

## Introduction

The COVID-19 pandemic is occurring in a new technological and social context, with an apparently endless and immediate access to the internet (Chen et al. 2020; Guessoum et al. 2020; Moreno et al. 2020). Health care professionals have thus raised concerns especially regarding problematic media use in children and adolescents (King et al. 2020; Király et al. 2020; Ko et al. 2020; Montag et al. 2020). In the general population, a dramatic increase of media use in adolescents is already reported (Thomasius 2020). However, the specific consequences for children and adolescents with Attention Deficit Hyperactivity Disorder (ADHD) in particular (Cortese et al. 2020) and on their media usage are not well known.

First publications on general mental and behavioral consequences of the pandemic have reported that under lockdown, children and adolescents with ADHD showed inflated conduct problems (Nonweiler et al. 2020), problems with remote learning (Becker et al. 2020), often worsened (Zhang et al. 2020), but sometimes also improved behavioral symptoms (Bobo et al. 2020). An Australian study reported increased media usage (gaming, TV, social media) along with positive and negative life style changes under lockdown for children and adolescents with ADHD (Sciberras et al. 2020). To which extent these changes may be permanent or transitory after the release of measures has not been investigated before.

In the first part of this study, a short introduction to aspects of problematic use of the internet (PUI) is given, with a focus on their relevance for ADHD. ADHD is considered to be one of the main comorbidities of PUI (Ho et al. 2014); inversely, ADHD is associated with a higher prevalence of PUI (Chen et al. 2015; Bioulac et al. 2008; Enagandula et al. 2018; Ha et al. 2006; Wang et al. 2017). In the second part of this publication, a survey with parents of patients referred for ADHD to child and adolescent psychiatry will be presented. In this survey, started after the spring lockdown 2020, we asked about changes in media time and problematic internet behaviors before, during and after the lockdown.

### Problematic internet use and ADHD

Problematic internet use (PUI) represents a broader, multifaceted field of problems related to internet use with similarity to impulse control disorders (Carli et al. 2013; Ioannidis et al. 2018; Shapira et al. 2000; Young 1998b). It refers to the inability to control one’s usage of the internet, resulting in dysfunctional patterns (e.g., excessive gaming, excessive use of social networks, excessive viewing of video clips etc.) with major negative consequences for interpersonal relationships, academic performance, and emotional well-being (Kubey et al. 2001; Liu et al. 2007; Spada 2014; Young 1998a; Yu et al. 2013). Besides excessive use and addictive aspects, PUI is also linked to other risky online behaviors, such as cyberbullying (Gámez-Guadix et al. 2016; Machimbarrena et al. 2018). Despite the evolving body of research on PUI, consensus on diagnostic criteria is lacking (Christakis et al. 2009; Ioannidis et al. 2018; Ko et al. 2005; Shapira et al. 2003), leading to inconsistencies in reported prevalence rates of PUI, ranging from 1 to 38% (Leung 2004; Rehbein et al. 2015; Vigna-Taglianti et al. 2017). An association between PUI and ADHD (Restrepo et al. 2020) or elevated media use and ADHD-related behaviors, respectively, has been frequently reported (e.g., Ceranoglu 2018; Nikkelen et al. 2014; Wang et al. 2017). The most consistent associations between ADHD symptoms and PUI have been found with impulsivity, and less with inattention and hyperactivity (El Asam et al. 2019; Kaess et al. 2014; Rosen et al. 2014).

### (Internet) Gaming disorder and ADHD

Gaming is considered as a continuum from harmless leisure activity to excessive use, with impairment and distress resulting in pathological or addiction-like behavior (Grüsser et al. 2006; Paulus et al. 2018). Gaming disorder (GD), as the best-studied domain in addictive online behaviors (Fauth-Bühler et al. 2017), has recently been included in the ICD-11 (King et al. 2019; WHO 2016; Yao et al. 2017). Internet Gaming Disorder (IGD) has been included in the DSM-5 (APA 2013), with diagnostic criteria matching those for substance use disorders (APA 2013; Petry et al. 2014; Rehbein et al. 2015). Prevalence rates of IGD range between 1 and 15%, depending on sample characteristics, age and country (Durkee et al. 2012; Gentile et al. 2017; Paulus et al. 2018). Individuals with IGD/GD share neurobiological alterations typical for ADHD, such as in brain regions associated with dopamine-mediated reward mechanisms (Weinstein et al. 2020), reduced activity in impulse control areas, and dysfunction in the prefrontal cortex (Brand et al. 2016; Rubia 2018).

Clinical or epidemiological studies have reported associations between IGD/ GD and ADHD symptoms (Stavropoulos et al. 2019), and in children and adolescents the severity of ADHD symptoms has been shown to correlate with problematic gaming (Chan et al. 2006; Paulus et al. 2018). Although addiction-like behaviors in PUI are usually linked to impulsivity in the literature (El Asam et al. 2019; Kaess et al. 2014; Rosen et al. 2014), a recent meta-analysis reported a stronger association between problematic gaming and inattention symptoms in ADHD (Mazurek et al. 2013). In sum, ADHD is both a comorbidity of IGD/GD and a risk factor for IGD/ GD (Ko et al. 2009) or problematic gaming (Carli et al. 2013). It has been stated that gaming could be a form of self-medication (Han et al. 2009), and a mean for coping with stress (Jones et al. 2014), or for escaping from everyday problems and distress (Snodgrass et al. 2014), which may contribute to the high rate of gaming in individuals with ADHD.

### Social media use and ADHD

Due to its high prevalence, social media use (SMU) is considered as normative behavior (Settanni et al. 2018). Addictive SMU, in contrast, is defined as a compulsive need and excessive preoccupation with social media, while other aspects of life such as relationships, school, and mental health related behaviors (sports, eating regularly and balanced, day–night rhythm including adequate sleep duration) are neglected and impaired [e.g., (Frost et al. 2017; Karaiskos et al. 2010; Lenhart et al. 2010)]. Prevalence among adolescents estimates vary, ranging from 4.5 to 43.2% (Bányai et al. 2017; Gul et al. 2018; Van Den Eijnden et al. 2016), with a higher prevalence in girls (Rumpf et al. 2014; Andreassen et al. 2012) and with growing mental health problems (Mérelle et al. 2017; Rosenberg et al. 2014).

Since SMU exerts positive short-term effects on mood, it is not surprising that adolescents with ADHD are more prone to SMU addiction (Barry et al. 2017) than non-affected controls. In addition, adolescents with ADHD may be especially attracted to SMU as they are more sensitive to external distractors (Cassuto et al. 2013). Students with higher ADHD symptoms are more likely to report high levels of addictive Facebook use (Settanni et al. 2018) and inversely, elevated SMU may also increase ADHD symptoms in adolescents (Boer et al. 2020; Cabral 2008).

### Cyberbullying and ADHD

Cyberbullying is defined as an intentional act to use electronic communication to embarrass or humiliate another person (Heiman et al. 2015; Kowalski et al. 2012; Kowalski et al. 2013). Higher rates of cyberbullying seem to result from difficulties with social interaction and impulsive and aggressive behavior, which is often seen in individuals with ADHD (Harty et al. 2009; Tokunaga 2010). Due to impaired or less developed social skills in individuals with ADHD (Shea et al. 2003), internet and social media provides a potential space for social contacts with peers while simultaneously likely leading to cyberbullying (Didden et al. 2009). Thus, individuals with ADHD seem to be at increased risk for cyberbullying as perpetrators (boys) or victims (girls) (Bacchini et al. 2008; Dawson et al. 2019; Gorucu Aydin et al. 2020).

### Problematic internet use, ADHD and therapy

To date, relatively few studies have investigated different treatment options for PUI and related disorders, even less for comorbid PUI and ADHD [e.g., (Chang et al. 2020)]. Although IGD is considered a treatable disorder, strong conclusions regarding treatment efficacy are lacking (Zajac et al. 2020). Cognitive behavioral therapy has received the largest evidence base for PUI (Spada 2014; Young 2007). Pharmaceutical agents such as methylphenidate (Han et al. 2009), escitalopram (Dell'Osso et al. 2008), citalopram (Sattar et al. 2004) and olanzapine (McElroy et al. 2008) or bupropion (Zajac, Ginley, and Chang 2020) have also been tested with some positive findings, but cognitive–behavioral therapy is established as the first choice therapy for internet addiction (Young 2011) or IGD (Stevens et al. 2019).

### Problematic internet use (PUI) in adults with ADHD

Most studies on PUI in adult ADHD have targeted young adults, often college students, in Asia, especially China, South Korea and Japan, where the prevalence of PUI is known to be much higher than in Western countries (Darvesh et al. 2020; Stavropoulos et al. 2020). A meta-analysis (Wang et al. 2017) reports similar prevalence rates of ADHD in adolescents (< 18 years) or young adults (> 18 years) with internet addiction. In a large scale survey with Chinese college students (aged 17–25 years), the total prevalence of internet addiction was 7.21% in male and 8.17% in females, and ADHD was a risk factor both for male and female internet addiction (male OR 6.487, female OR 4.497) (Shen et al. 2020). The association between ADHD characteristics and PUI has also been reported in others cultures: In a large cross sectional study with over 22,000 adult participants (mean age 35.8 years) (Andreassen et al. 2016), ADHD was stronger positively associated with addictive social networking than with online gaming, and both were related to lower adult age. Other studies also point towards an association between PUI, ADHD symptoms and lower adult age, e.g., between smartphone addiction and ADHD inattention symptoms (Panagiotidi et al. 2018), while PUI in older adults seems more often related to OCD or depression (Ioannidis et al. 2018).

## Online survey on screen media use before, during and after the COVID-19 lockdown

### Goals of the study

In the following section, the results of an online survey conducted with parents of children and adolescents referred to child and adolescent psychiatry for ADHD are reported. Patients with ADHD are known to be particularly at risk for PUI. The goals of the study were to investigate (1) the impact of the lockdown on media use, especially on media time, as well as on risky online behaviors in patients referred for ADHD to child and adolescent psychiatry. (2) We wanted to investigate possible associations between the use of digital media and behavioral or emotional problems under the lockdown. (3) Finally, we intended to investigate the course of media-related behavior after the release of measures. Although we hypothesized to find a sizable increase of media time under the lockdown, we could not project whether we had to expect a chronification of media overuse, such as apprehended by some mental health specialists [e.g., (Király et al. 2020)], or would rather find a “back to normal” after the release of measures.

### Method

#### Recruitment, procedure and timeline

Parents of patients aged between 10 and 18 years, who had been referred during the last 2 years to an outpatient clinic of the Department of Child and Adolescent Psychiatry and Psychotherapy (CAPP), University of Zurich, Switzerland, were invited to participate in an anonymous online survey. Invitations to participate and a link to access the survey were sent to parents of all patients of the defined time frame by email. The questions were to be completed by one parent. Data collection started in the last week of May 2020 and ended in the first week of July 2020. In Switzerland, a complete lockdown with homeschooling and onsite school closure began on March 16th, and a first easing of measures occurred on the 26th April. The first schools reopened on May 11th. At the time of the survey, in June 2020, most students had returned to classes, at least part time (Table 1).

**Table 1:** Sample description: demographics and comorbidities

	*N*	%	Age mean	SD	Age range	Ratio m/f
All	126	100	13.21	2.29	10–18	94/32
Age ≤ 13 years	66	52.4%	11.31	1.09	10–13	47/19
Age ≥ 14 years	60	47.6%	15.32	1.21	14–18	47/13
Male	94	74.6%	13.14	2.37	10–18	
Female	32	25.4%	13.50	2.17	10–18	
Comorbidities						
None	75	50.5%	13.27	2.29	10–18	59/16
Learning disorder	20	15.9%	12.86	2.47	10–16	13/7
Depression	6	4.7%	14.17	2.48	11–17	4/2
Anxiety	5	4.0%	11.00	1.22	10–13	4/1
Eating disorder	4	3.2%	15.25	2.06	13–17	1/3
Other	16	12.7%	13.90	2.33	10–18	13/3

School						
School situation of the last 2 weeks	Same as January 2020	Single days/part time at school	Online, some hours	Online, regular school hours	Other/ not applicable	
% Responses	56.3%	37.3%	4.0%	1.6%	0.8	

SD = standard deviation. Deviations from 100% may be due to rounding

The study was conducted in accordance with the principles of the declaration of Helsinki and with the regularities of the local Ethics committee.

#### Instruments

The survey consisted of two parts: an adapted version of a new PUI-screening (“PUI-Screening Questionnaire for Children and Adolescents” (PUI-SQ); Werling et al., submitted) developed for the routine screening of PUI in child and adolescent psychiatry, and the European CoRonavIruS Health Impact Survey 3.2 (CRISIS) of the ECNP group, which was developed to assess mental health problems and well-being of patients and their parents during the COVID-19 crisis, and will be analyzed elsewhere. The present study focuses on screen media related behavior assessed by the PUI-SQ. However, basic information on demographic data, psychopathological symptoms and PUI-relevant aspects of psychological well-being were drawn from the CRISIS 3.2 questionnaire.

The PUI-SQ was conceived as a paper-and-pencil instrument for problematic internet use and screen-media related problems in clinically referred children and adolescents. We present the results of a shortened version comprising the following subscales:

- Negative impact subscale: Impact of media use on everyday life and addiction-like behaviors (seven items)
- Digital risks and problems subscale: Parents’ concern about problem behavior and risks related to media use (eight items)
- Media time: Amount of time per day (during leisure time) spent on different digital activities (gaming, social media) and with different devices (mobile/smartphone, PC/tablet, gaming console, TV) (six items)

For the present study, each item had to be rated three times on a 4-point Likert scale (from “not true” to “absolutely true”): regarding the behavior (1) retrospectively before the Corona crisis (Time 1: January/February 2020), (2) during the lockdown (Time 2: March/April 2020), and 3. after the lockdown (Time 3: “last two weeks”, May/June2020). The estimated media time was indicated on a 5-point rating scale (“no time”, “less than 1 h”, 1–3 h”, 4–6 h”, “more than 6 h”).

#### Statistical analysis

Parents’ item responses were analyzed descriptively. Interval scaled data were analyzed by ANOVA (or repeated measures ANOVA). For PUI-SQ subscales 2 and 3, scores were calculated. For statistical comparison, responses on time duration spent with media were recoded using the medium value of each time range category as a numerical value (i.e., ½ h, 2 h, 5 h, and 7 h; the latter as a conservative estimate for the category > 6 h). Estimated total media time was calculated by adding up the time durations for all devices (smartphone, tablet/PC, game console, TV). When distributional assumptions were not met, ANOVAs, which are known to be robust against violations (Blanca et al. 2017), were nevertheless used, Greenhouse–Geisser corrections were reported and post-hoc pairwise analyses were performed by nonparametric tests and Bonferroni-corrected.

### Results

#### Participants

The present sample of *N* = 126 patients referred for ADHD to the Department of child and adolescent psychiatry (CAPP) is drawn from a larger sample of patients with all types of psychopathologies, whose parents responded to the survey. The response rate of all parents was approximately 28%. All information was collected anonymously and diagnostic classification was based entirely on self-assessments: Parents were asked to indicate the main psychopathological problem for which their child had been referred to the CAPP, by selecting among psychopathological categories or by specifying the nature of the problem in a free text section. Parents could indicate a second problem in a subsequent question. The present sample includes participants who indicated ADHD as the main psychopathological problem (Table 1). Patients with autism spectrum disorder as second problem were excluded. Girls were underrepresented in this sample with a ratio of 1:3, which is approximately representative of the ADHD gender distribution in a clinically referred population at this age.

#### Time spent with media

**Fig. 1:**
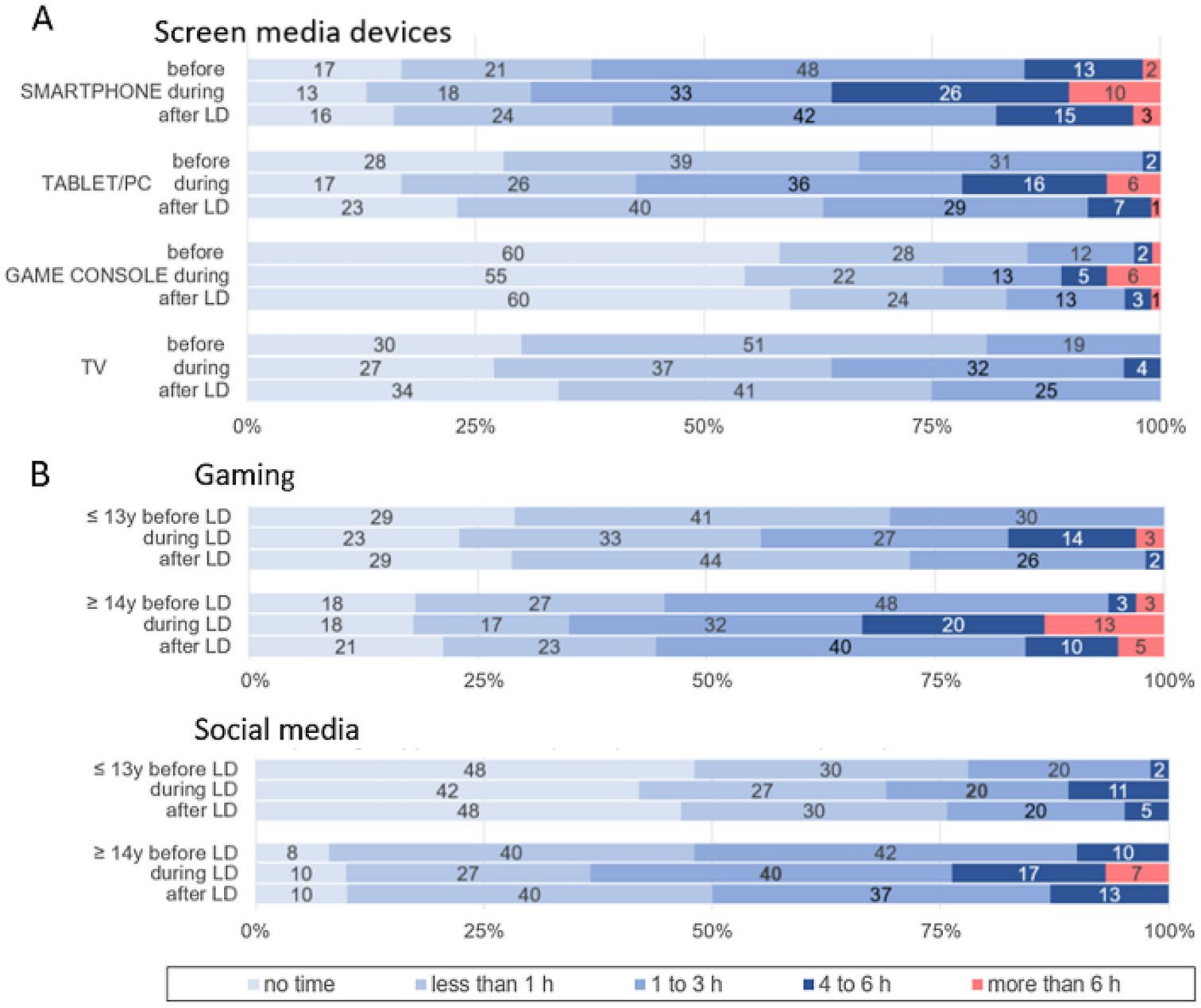
Hours per day during leisure time spent with screen media/the internet before the Corona crisis (before LD), during lockdown (during LD) and after lockdown (after LD = “last 2 weeks”). **a** Screen media devices: Hours per day spent with smartphone, tablet/PC, game console or TV (*N* = 126). **b** Digital Activities: Hours per day spent with gaming and social media in children (10–13 years, *N* = 66) and adolescents (14–18 years, *N* = 60). Numbers refer to percent of responses

Media consumption increased considerably during the lockdown (Fig. 1a). Smartphone use over 4 h per day increased from 15% of patients before the Corona crisis to 36% under lockdown, tablet/PC use from 2 to 22% and gaming console use from 3 to 11%. Excessive TV use under lockdown (over 6 h) was not reported, but a small group (4%) began to watch TV for more than 4 h per day. Adolescents (14–18 years) spent considerably more time on gaming and with social media under lockdown than did children (10–13 years) (Fig. 1b). The percentage of patients who spent more than 4 h on gaming increased from 6 to 23% in adolescents and from 0 to 17% in children. Likewise, the proportion of social media time use over 4 h increased from 10 to 24% in adolescents and from 2 to 11% in children.

It is worthy of mention that girls showed a slightly different pattern of use, with high social media time (over 4 h: 28%, *N* = 9) but low gaming time (over 4 h: 3%, *N* = 1; no gaming: 53%, *N* = 17) under lockdown. However, due to the small number of female patients, we did not analyze gender differences further.

**Fig. 2:**
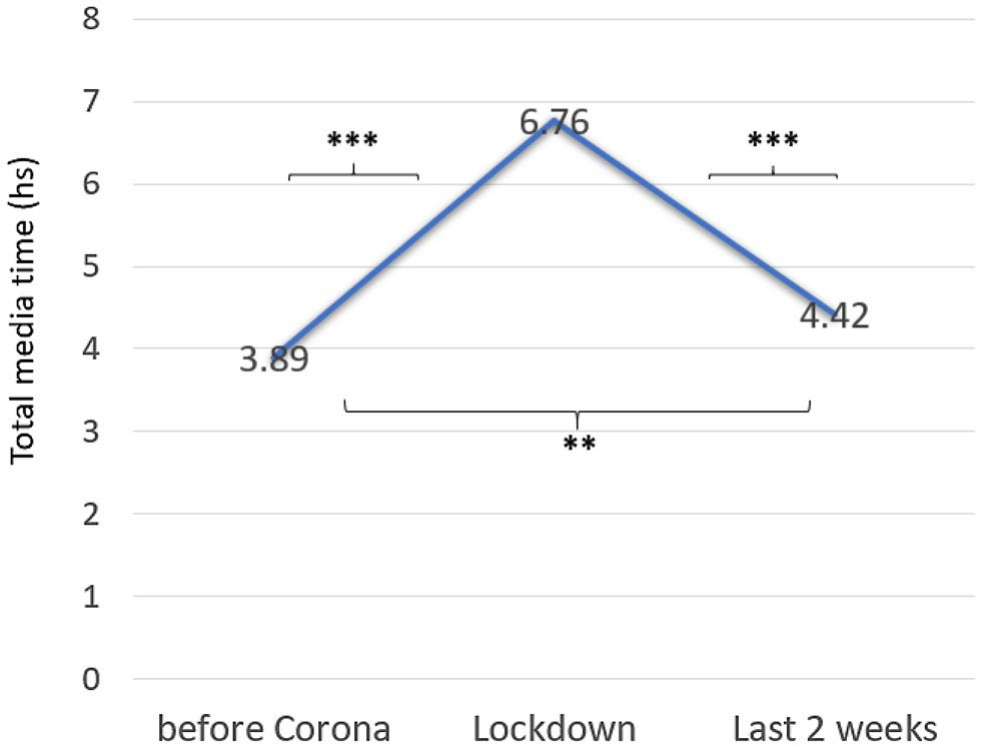
Mean estimated total media time (hours) in patients with ADHD before, during and after the lockdown (*N* = 126). **p < 0.01, ***p < 0.001

The comparison of estimated total media time (eTMT) over time for the full group, resulted in a significant increase from pre-Corona to lockdown (T1–T2), followed by a significant decrease after the easing of lockdown measures (“last 2 weeks”) (T2–T3) (Fig. 2) (*F*(1.425,179) = 81.144, *p* < 0.001). Nevertheless, eTMT did not completely return to pre-Corona levels at T3 ((*Z* = − 3.448), *p* < 0.001), but remained significantly higher (Fig. 2). In the full group, repeated measures analyses did not reveal different effects or interactions regarding age groups over time (*F*(1.425,179) = 2.272, *p* < 0.122), while between-group effects showed that adolescents had considerably higher eTMT than did children (*F*(1,124) = 22.245, *p* < 0.001) (during lockdown: adolescents mean eTMT = 8.39 h, children mean eTMT = 5.29 h).

#### Negative impact of media use before and under lockdown

A moderate to severe negative impact of screen media use on everyday life aspects before Corona was reported by 15% (mental health) to 32% (family life) of parents (Table 2). Under lockdown, the negative impact on family life increased in 29%, with a total of 46% of parents reporting a major negative impact (30.2% slightly true, 15.9% absolutely true). Moderate to high concern about aggressive reactions and secret media use were already very common before Corona and these concerns increased relatively little under lockdown: from 46 to 50% of parents who described a major concern about aggressiveness, and from 34 to 40.5% about secret media use. 6.3% of parents stated that they had no agreement with their child regarding media time, which may be explained by the high mean age in this small group (*N* = 8, mean age 16.0 years). The negative impact subscale score increased significantly from pre-Corona (T1) (mean 13.90, SD 4.7) to lockdown (T2) (mean 15.38, SD 5.83; T1:T2 *Z* = − 3.819, *p* < 0.001), and subsequently decreased from lockdown (T2) to “last 2 weeks” (T3: mean 14.34, SD 5.47, *Z* = − 3.81, *p* < 0.001). Differences between T1 and T3 were only not significant anymore after Bonferroni correction (*α* = 0.016; *Z* = − 2.356, *p* < 0.018). Thus, taken together, it can be concluded that negative effects of screen media use on everyday life significantly increased under lockdown, but these effects were mostly reversible and returned in part to pre-Corona values once lockdown measures were eased.

**Table 2:** Negative impact of media use before and during the lockdown

	Before Corona (T1)				Lockdown (T2) compared to T1		Last 2 weeks (T3) compared to T2	
Negative impact of child’s media use on…	Not true	Slightly true	Quite true	Absolutely true	Worse	Better	Better	Worse
…family life	20%	49%	27.0%	5%	29%	8.2%	23%	7%
…homework and academic achievements	34%	42%	20%	4%	26%	9%	18%	8%
…friendships and social activities in real life	52%	33%	14%	2%	23%	5%	18%	6%
…mental well-being and mental health (e.g., mood)	36%	48%	14%	1%	18%	6%	14%	6%
…physical well-being and health (e.g., sleep)	45%	36%	18%	2%	23.6	7.0%	14.9%	6.5%

	Before Corona (T1)				Lockdown (T2) compared to T1		Last 2 weeks (T3) compared to T2	
I am concerned because my child …	Not true	Slightly true	Quite true	Absolutely true	Concern		Concern	
					More	Less	More	Less
…becomes aggressive/very angry when media use is restricted	23%	31%	32%	14%	19%	6%	5%	16%
…secretly spends more time with media than agreed upon^a^	32%	29%	24%	10%	28%	7%	5%	19%

^a^Not applicable/no agreement was made between parents and child: 6%

#### Specific risks and problems

On the specific problem and risks subscale, the majority of parents indicated no or only minor concern (Table 3). A comparison of pre-Corona and lockdown subscale scores did not yield significant differences (T1 mean = 10.01, SD 3.0; T2 mean = 10.11, SD 3.2; *Z* = − 0.960; *p* < 0.339), demonstrating that specific or risky problem behavior was not induced or changed by the lockdown situation. Therefore, we only report lockdown results here (Table 3). Adolescents’ subscale scores were significantly higher than those of children (10.66, SD 2.80 vs. 9.60, SD 3.49; *Z* = − 2.919, *p* < 0.004). with the largest differences between adolescents and children being found regarding a major concern (responses slightly true and absolutely true) about the use of problematic or violent video games (13.3% compared to 6%) and the careless or risky handling of personal data and.information (16.6% compared to 4.5%). In both age groups, the frequency of major concern about cyberbullying was low (5% vs. 7.6%).

**Table 3:** Problem behaviors and media related risks under lockdown in children (≤ 13 years) and adolescents (≥ 14 years)

	Children (*N* = 66) (= 100%)				Adolescents (*N* = 60) (= 100%)			
I am concerned that my child might…	Not true	Slightly true	Quite true	Absolutely true	Not true	Slightly true	Quite true	Absolutely true
….be a victim of cyberbullying	77.3	15.2	6.1	1.5	71.7	23.3	5.0	0.0
… be a cyberbullying perpetrator	87.9	9.1	1.5	1.5	83.3	16.7	0.0	0.0
… be too careless with risks on the internet (e.g., handling of personel data, photos)	57.6	37.9	3.0	1.5	56.7	26.7	13.3	3.3
… play video games with harmful or age-inappropriate content (e.g., trivializing violence)	63.6	30.3	4.5	1.5	38.3	48.8	8.3	5.0
…visit problematic chatrooms / chat groups (e.g., promoting self-harm)	80.3	13.3	3.0	3.0	60.0	31.7	8.3	0.0
…watch films, series or clips with harmful or age-inappropriate content	48.5	37.9	9.1	4.5	30.0	56.7	11.7	1.7
… illegally download or download or distribute prohibited content	83.3	13.6	1.5	0	61.7	36.7	1.7	0

#### Relation between total media time and ADHD related problems under lockdown

**Fig. 3:**
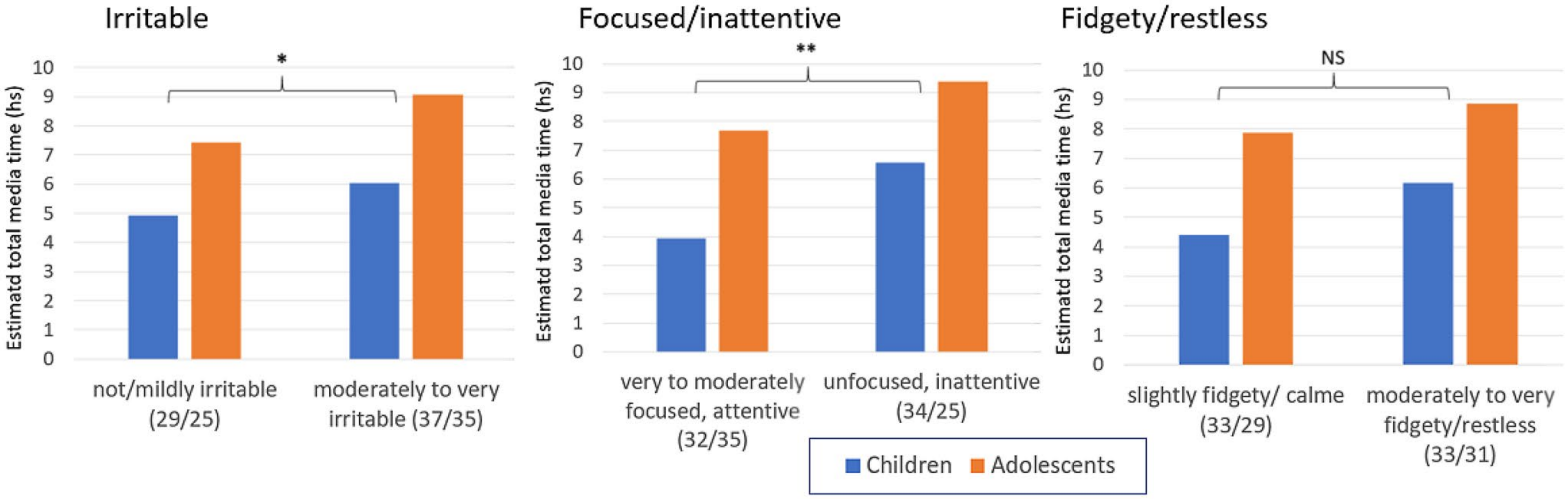
Estimated total media time (hours) in subgroups of children and adolescents with higher versus lower irritability, attention/inattention, and hyperactivity/restlessness during the lockdown as rated by parents. Adolescents had significantly higher media times, but age- by subgroup interactions were not significant. Numbers in parentheses = *N* (children/adolescents). *p < 0.05; **p < 0.01, *NS* non significant

Four items of the CRISIS 3.2 questionnaire were directly be related to ADHD symptoms: an item asking parents whether the main psychopathological problem (that is ADHD) had changed, and three items asking about irritable, hyperactive/restless or unfocused/unconcentrated behavior during the lockdown. All answers were rated on 5-point Likert scales. We investigated whether these ratings of symptoms characteristic for ADHD could be related to the total time spent with media under lockdown. 46.8% of parents (*N* = 59) reported no change of the main psychopathological problem, 33.3% (*N* = 42) an improvement and 19.8% (*N* = 25) a deterioration. Patients with a deterioration of ADHD symptoms had significantly longer media times (eTMT = 9.5 h, SD 5.23) than those with an improvement (eTMT = 4.80 h, SD = 2.72, *Z* = − 4.020, *p* < 0.001). Patients who had been rated as moderately, quite or very irritable (*N* = 72) had more elevated eTMT than those who were rated as slightly/not irritable (*F*(2/120) = 3.236, *p* < 0.043). Similarly, patients with poor or very poor ability to focus/concentrate under lockdown (*N* = 59) had higher eTMT than patients with moderate or good concentration (*N* = 67) (*F*(1/122) = 7.952, *p* < 0.006). However, patients with more (*N* = 62) or less (*N* = 64) hyperactivity/restlessness did not significantly differ regarding eTMT (*F*(1.122) = 3.161, *p* < 0.078) (Fig. 3).

### Discussion

In this sample of patients clinically referred for ADHD, a 42% increase in total media time was observed under lockdown according to parents’ ratings. After the lockdown, i.e., after the return to school for most students, total media time decreased considerably, but did not completely return to pre-Corona levels. Parents reported a significant increase of negative effects of media use on everyday life under lockdown, especially on family life, but this effect was rather not permanent. Other media related problems and risks behaviors such as cyberbullying or careless handling of personal data were rarely reported and did not substantially increase under lockdown. Elevated media time under lockdown was found in patient subgroups with high irritability, concentration problems and a deterioration of ADHD symptoms. This is in line with the reviewed literature and supports the association between ADHD traits and the risk of excessive media use (Carli et al. 2013; Dullur et al. 2021; Stavropoulos et al. 2019; Wang et al. 2017). Nevertheless, a higher number of parents reported an improvement rather than deterioration of ADHD-related problems during the lockdown. This rather surprising finding was also observed by others (Bobo et al. 2020), and was explained by the reduction of school- and peer-related stress during the lockdown.

The number of patients with excessive gaming or other excessive media use (more than 6 h a day) was not higher than in studies on media use of the general population in German speaking countries (e.g., (Suter et al. 2018), although this might have been expected in a population at risk. One explanation is that the present studied sample probably cannot be considered as representative of the full range of patients with ADHD, but only of those whose parents were willing and able to complete an anonymous survey on media and COVID-19 without any incentives. In addition, while most surveys on adolescents’ media use are based on self-reports, the estimation of media time in the present study are based on parent ratings, which may lead to an underestimation.

### Limitations

Some limitations of the study should be mentioned: first, all ratings of change are based on parents’ retrospective evaluations and may, therefore, be biased. Second, we did not collect objective data on socio-economic status and cultural background. Parents indicated that their child had been referred to child and adolescent psychiatry for ADHD, but for a minority still in the diagnostic process, the diagnosis may not have been confirmed. Finally, all diagnostic categories were self-assessed.

### Conclusion

As shown in the short literature summary, children, adolescents and young adults with ADHD are at risk of PUI. According to the present study on screen media use before, during and after the lockdown in spring 2020, total media time considerably increased during the lockdown in a sample of children and adolescents referred for ADHD to child and adolescent psychiatry, subsequently decreased after the easing of measures, but remained at a higher level than before the crisis. To the best of our knowledge, this is the first study to compare media related behavior before, during and after the lockdown in children and adolescents clinically referred for ADHD.
